# Supplementary material for: Genomic comparison and phenotypic characterization of Pseudomonas aeruginosa isolates across environmental and diverse clinical isolation sites
Source: mSystems. 2026 Feb 5;11(3):e01362-25. doi: 10.1128/msystems.01362-25 (PMC13011455; doi:10.1128/msystems.01362-25)

Genomic comparison and phenotypic characterization of *Pseudomonas aeruginosa* isolates across environmental and diverse clinical isolation sites

Cristina Penaranda[1,2,3,4,#,^], Evan P Brenner[5,^], Anne E Clatworthy[3,4], Lisa A Cosimi[4], Janani Ravi[5,#], Deborah T Hung[3,4,6,#].

[1]Department of Immunology and Genomic Medicine, National Jewish Health, Denver, CO. [2]Department of Immunology and Microbiology, University of Colorado Anschutz, Aurora, CO. [3]Department of Molecular Biology and Center for Computational and Integrative Biology, Massachusetts General Hospital, Boston, MA. [4]Infectious Disease and Microbiome Program, Broad Institute of Harvard and MIT, Cambridge, MA. [5]Department of Biomedical Informatics, University of Colorado Anschutz, Aurora, CO. [6]Department of Genetics, Harvard Medical School, Boston, MA. [^]Co-primary authors; [#]Corresponding authors.

Supplemental figures

Supplemental Figure 1. Genome assembly and annotation statistics.

All whole genome sequencing data were cleaned and checked for quality using FASTP, assembled *de novo* using Shovill, and annotated using Bakta. Summary statistics overall and per isolation source are provided.

Supplemental Figure 2. Clade classification of *Pseudomonas aeruginosa* strains.

A. A SNP-based phylogenetic tree rooted on the phylogenetic outlier PA7 in comparison with the strains used in this study. **B**. Clade distribution for each isolation source. **C.** Pairwise Fisher's Exact Test results for clade distribution. Environmental strains were overrepresented in Group A, while urine and eye strains were overrepresented in Group B.

Supplemental Figure 3. Correlation of clade and virulence phenotypes.

Virulence phenotypes were compared between Group A (n=92) and Group B (n=34) strains. Biofilm formation and cytotoxicity (decreased host cell survival) were significantly greater in Group B strains. Wilcoxon test with Benjamini-Hochberg correction for multiple testing: $^{**}p<0.01$; $^{***}p<0.001$.

Supplemental Figure 4. Antibiotic Susceptibility Testing Phenotypes.

Antibiotic Susceptibility Testing phenotype for each strain-antibiotic combination based on the Zone Diameter Interpretive Chart for *P. aeruginosa* reference strain ATCC 27853.

Supplemental Figure 5. Non-cytotoxic Group B strains.

A. A heatmap showing the pairwise SNP distance between Group B strains. **B.** The BL18 *exoU* allele shows reduced cytotoxicity compared to the PA14 reference. Deletion of *exoU* from PA14 yields high host cell survival as expected, and overexpression from a plasmid vector with the

PA14 *exoU* allele restores wild-type toxicity. Expression of the BL18 *exoU* allele significantly increased host cell survival, supporting the *exoU* locus as responsible for the BL18 strain's attenuated host cell killing. **C.** SNPs in the BL18 *exoU* allele compared to PA14 yield a cluster of missense mutations in the C-terminal portion of the ExoU domain (Conserved Domain Database ID: cd07207). **D.** SNPs in BWH060 *exsA* allele compared to PA14 show a truncation with a premature stop at Q43. **E.** A missense SNP in the BWH038 *mucA* allele compared to PA14 results in a frameshift at A144.

Supplemental Figure 1

Genome assembly and annotation statistics

	Number of Isolates	Mean Length (±SD)	Length Distribution	Mean CDSs (±SD)	CDSs Distribution
**Overall summary statistics**					
Total	128	6.65 Mbp ± 0.28 Mbp	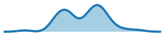	6074 ± 269	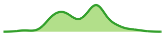
**Summary statistics by isolation source**					
blood	9	6.79 Mbp ± 0.15 Mbp	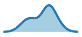	6212 ± 138	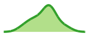
environmental	28	6.62 Mbp ± 0.28 Mbp	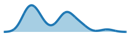	6050 ± 287	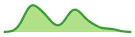
eye	27	6.75 Mbp ± 0.27 Mbp	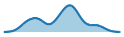	6150 ± 266	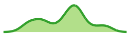
reference	3	6.40 Mbp ± 0.14 Mbp	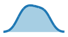	5830 ± 136	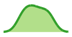
other	11	6.63 Mbp ± 0.23 Mbp	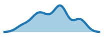	6080 ± 244	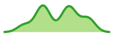
pulmonary	20	6.59 Mbp ± 0.30 Mbp	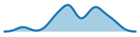	6014 ± 277	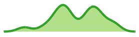
urine	22	6.64 Mbp ± 0.32 Mbp	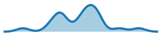	6048 ± 300	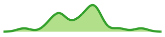
wound	8	6.60 Mbp ± 0.23 Mbp	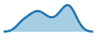	6043 ± 230	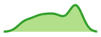

Supplemental Figure 2

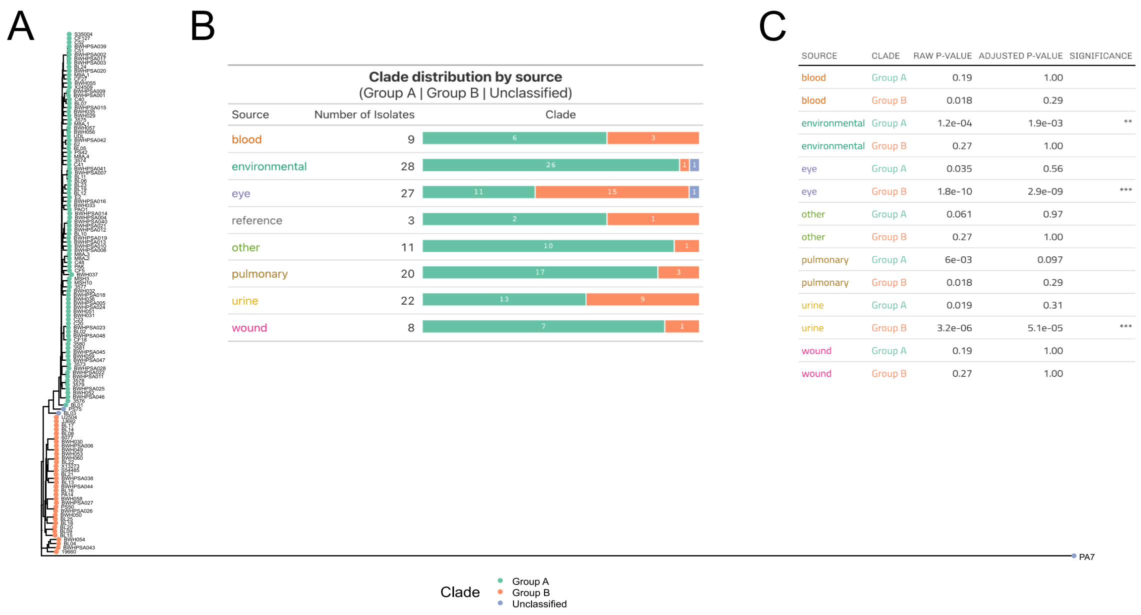

Clade distribution by source
(Group A | Group B | Unclassified)

Source	Number of Isolates	Clade
blood	9	6 | 3
environmental	28	26 | 1 | 1
eye	27	11 | 15 | 1
reference	3	2 | 1
other	11	10 | 1
pulmonary	20	17 | 3
urine	22	13 | 9
wound	8	7 | 1

SOURCE	CLADE	RAW P-VALUE	ADJUSTED P-VALUE	SIGNIFICANCE
blood	Group A	0.19	1.00	
blood	Group B	0.018	0.29	
environmental	Group A	1.2e-04	1.9e-03	**
environmental	Group B	0.27	1.00	
eye	Group A	0.035	0.56	
eye	Group B	1.8e-10	2.9e-09	***
other	Group A	0.061	0.97	
other	Group B	0.27	1.00	
pulmonary	Group A	6e-03	0.097	
pulmonary	Group B	0.018	0.29	
urine	Group A	0.019	0.31	
urine	Group B	3.2e-06	5.1e-05	***
wound	Group A	0.19	1.00	
wound	Group B	0.27	1.00	

Supplemental Figure 3

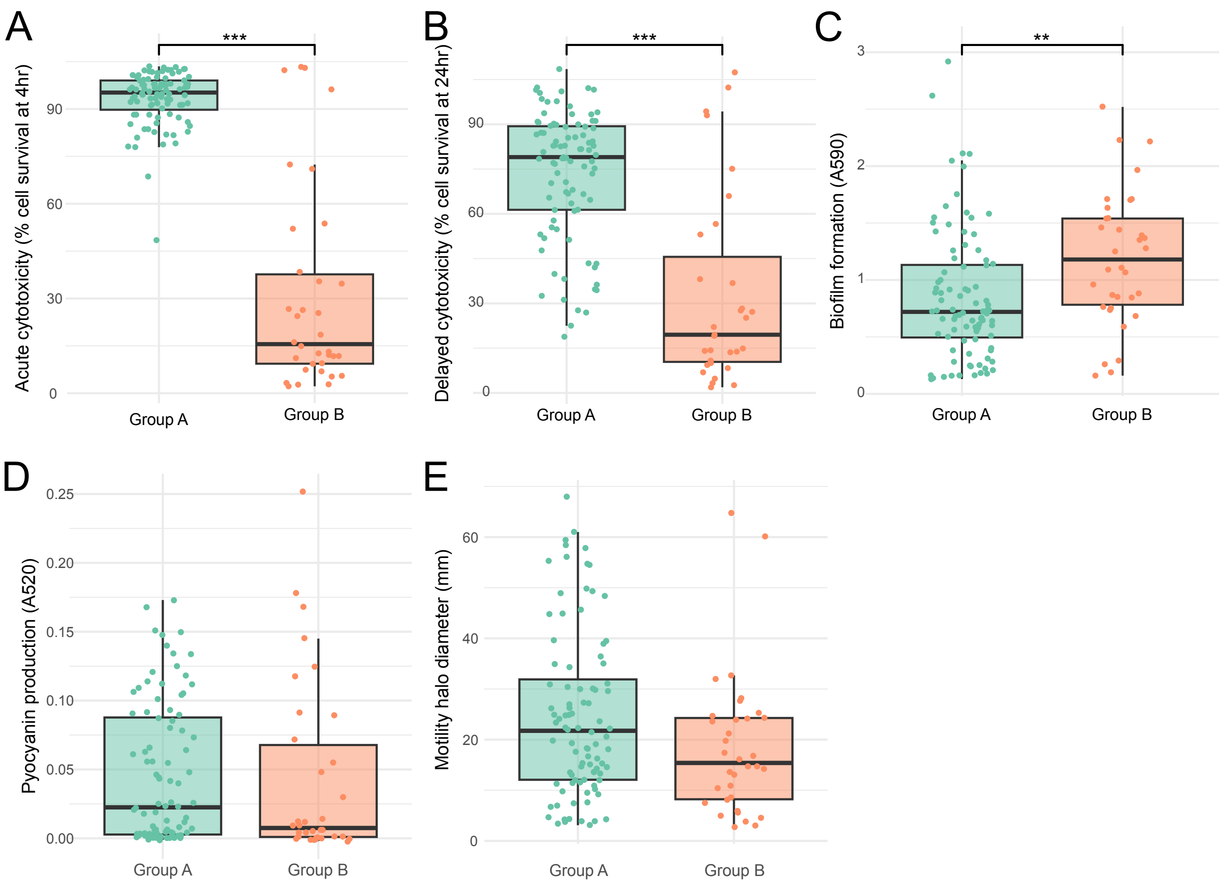

Supplemental Figure 4

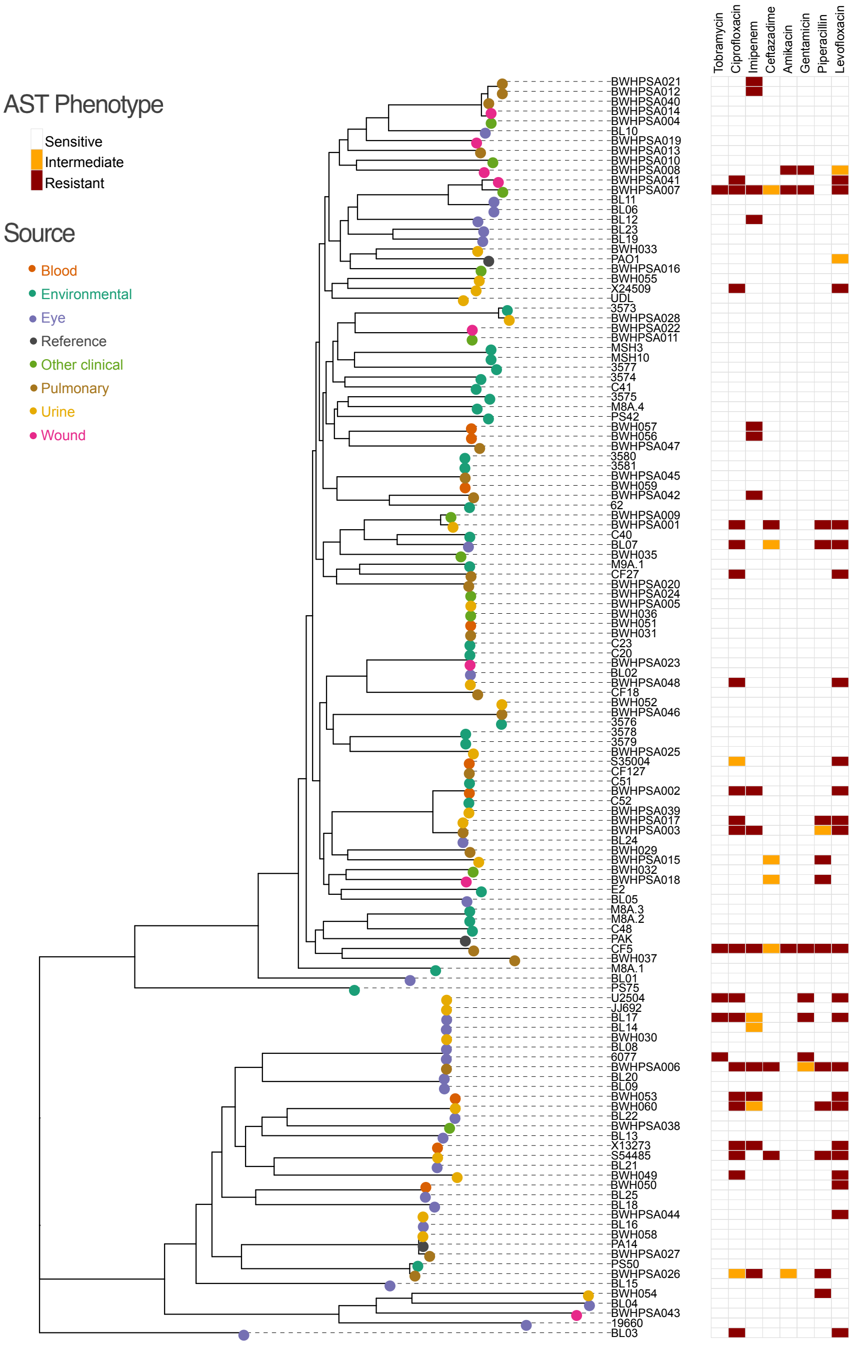

Supplemental Figure 5

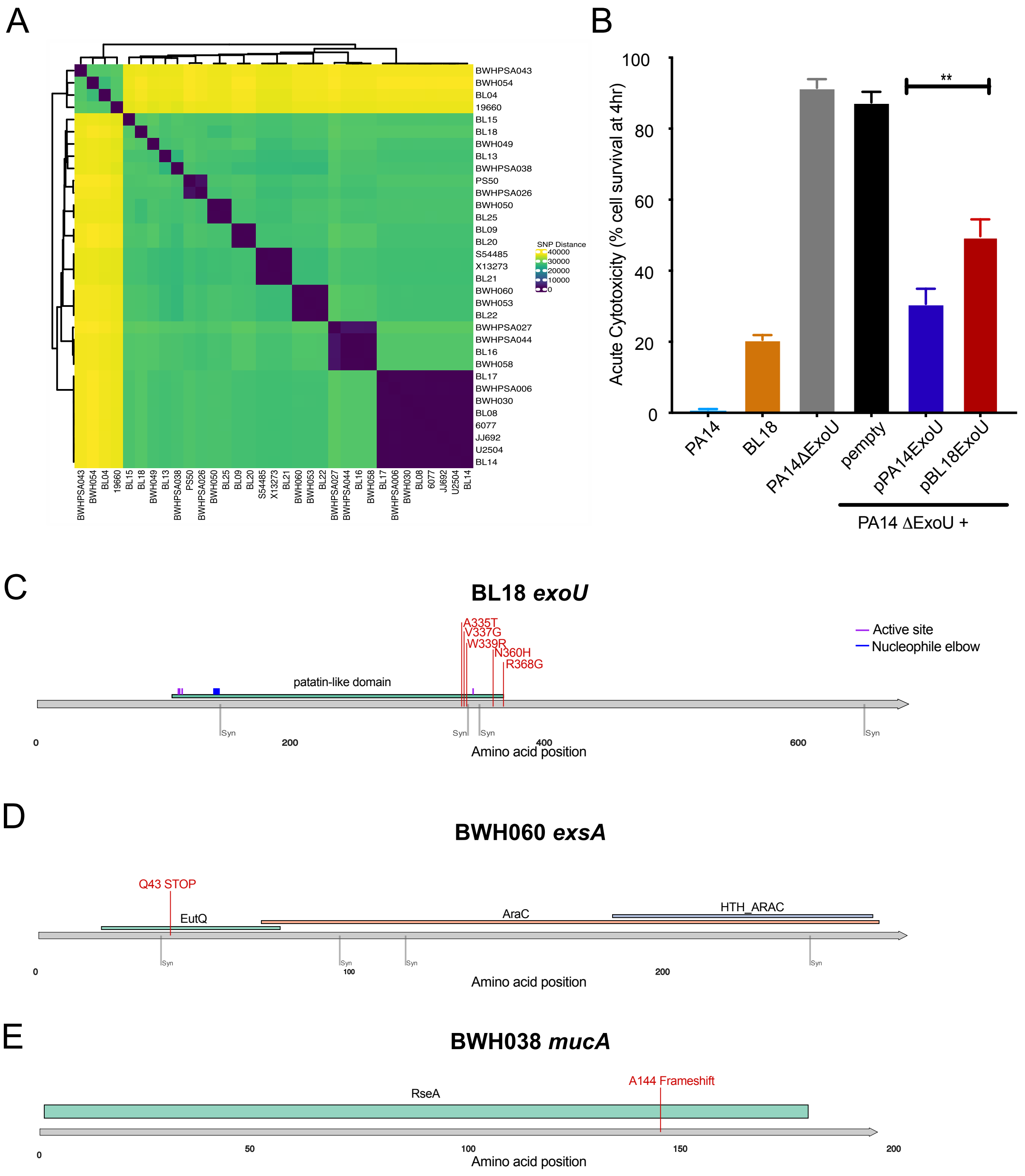

Supplement: Supplemental Figures — Figures S1-S5. [file msystems.01362-25-s0009.pdf]
