# Supplementary material for: Genomic comparison and phenotypic characterization of Pseudomonas aeruginosa isolates across environmental and diverse clinical isolation sites
Source: mSystems. 2026 Feb 5;11(3):e01362-25. doi: 10.1128/msystems.01362-25 (PMC13011455; doi:10.1128/msystems.01362-25)
Supplement: Captions — for Data S1-S8. [file msystems.01362-25-s0010.docx]

# Supplementary data

**Supplemental Data 1. Genome accession IDs and associated metadata.**

Sheet *SRA_IDs* contains the strain accession numbers and sequencing metadata for our 125 isolates. *Ref_Strain_IDs* lists the RefSeq accession numbers for laboratory reference strains used. *Strain_origins* shows the original isolation source metadata recorded (*PreciseSource*) as well as our fine classification (*Source*) and broad classification (*GroupedSource*). *Phenotypes* records the antimicrobial susceptibility testing results for tested compounds, plus the mean, standard deviation, and replicates for the virulence assays performed.

**Supplemental Data 2. Tree metrics.**

Sheet *ModelFinder_Results* shows the best performing substitution models as chosen by ModelFinder for our dataset. AIC and BIC yielded slightly different best models, and for our set we selected the top model based on BIC. *IQ-TREE2_ParameterResults* reports the initial and final model parameters for our phylogenetic estimation.

**Supplemental Data 3. Consensus phylogenetic tree with confidence estimates**

We provide the unmodified output consensus tree built using core SNPs with estimated confidence values from IQ-TREE2 as described above.

**Supplemental Data 4. Pangenomic associations with trait and isolation source.**

A microbial genome-wide association study was performed with Scoary2 to associate genes with virulence traits or isolation sources. As an example, *4hr_Cytotox_Scoary2* reports *exoU* as a top hit for the trait of host cell survival – that is, g- (lacking genotype *exoU*) is associated with t+ (trait of greater host cell survival). Few associations across all tests remained significant after controlling for multiple testing. Many genes have uninformative “group_####” names, indicating that consensus names were not identified during pangenome feature clustering. Representative protein sequences are provided for downstream investigation of all features.

**Supplemental Data 5. Sequences and polymorphisms for exoenzymes.**

*Summary_nonSYN* lists non-synonymous mutations in *exoU*, *exoT*, *exoS*, and *exoY*; WT indicates the same sequence as reference, “-” indicates the gene is not found in the genome*.* *exo_AllSNPs* lists a comprehensive set of all SNPs observed in all strains, while subsequent sheets are subsetted per-exoenzyme.

**Supplemental Data 6. Correlation analysis.**

We tested whether virulence traits were correlated among isolates from different sources or phylogenetic groups. For example, *GroupA_Subset* reports Spearman and linear regression modeling tests for correlation between different traits in Group A isolates. As expected, 4hr and 24hr host cell survival are significantly correlated, while most other traits are not strongly correlated.

**Supplemental Data 7. Computationally identified AMR mechanisms per strain.**

We used AMRFinderPlus to identify mechanisms for antimicrobial resistance in our strains. We listed features found in >50% of strains “Common_mechanisms,” and rarer mechanisms “Specific_mechanisms.” If explanatory mechanisms for resistance towards a given drug were identified, they are recorded as TRUE, and if specific mechanisms are lacking, FALSE is shown. If an isolate showed susceptibility for a given drug, the mechanistic explanation for that phenotype is simply reported as NA.

**Supplemental Data 8. Differentiating SNPs in strains PS50 and BWHPSA026.**

PS50 and BWHPSA026 showed stark cytotoxicity differences despite few genetic differences (132 missense variants in protein coding genes). PS50 was cytotoxic (25% host cell survival), while BWHPSA026 was not (100% host cell survival).
